# Lupus mastitis of the male breast

**DOI:** 10.1259/bjrcr.20150290

**Published:** 2016-05-30

**Authors:** Ajit Thapa, Anushri Parakh, Jyoti Arora, Ruchika Kumar Goel

**Affiliations:** ^1^ Department of Radiology and Imaging, Medanta – The Medicity, Gurgaon, India; ^2^ Clinic of Radiology and Nuclear Medicine, University Hospital of Basel, Basel, Switzerland; ^3^ Department of Pathology, Medanta – The Medicity, Gurgaon, India

## Abstract

A 39-year-old male with no known co-morbid conditions presented with gradually increasing bilateral breast lumps for 1.5 years. Clinically, tender subcutaneous masses were detected. Mammograms revealed masses on both sides that on ultrasound were hyperechoic and showed internal vascularity. An MRI was suggested to assess the extent of the disease that confirmed bilateral masses but was otherwise inconclusive. Core biopsy revealed evidence of panniculitis with likely autoimmune aetiology. Evaluation of autoimmune markers was carried out that was positive and multidisciplinary team discussion concluded the diagnosis as lupus mastitis. Male breast pathology and lupus mastitis are both uncommon conditions, making lupus mastitis of male breast an extremely unusual presentation. However, its close clinical and radiological similarity with malignancy makes it important in spite of its rarity. Here we report a case of bilateral lupus mastitis in male breast with its radiological features.

## Summary

A 39-year-old male with no known comorbid conditions presented with gradually increasing bilateral breast lumps for 1.5 years. Clinically, tender subcutaneous masses were detected. Mammograms revealed masses on both sides that on ultrasound were hyperechoic and showed internal vascularity. An MRI was suggested to assess the extent of the disease that confirmed bilateral masses but was otherwise inconclusive. Core biopsy revealed evidence of panniculitis with likely autoimmune aetiology. Evaluation of autoimmune markers was carried out that was positive and multidisciplinary team discussion concluded the diagnosis as lupus mastitis. Male breast pathology and lupus mastitis are both uncommon conditions, making lupus mastitis of male breast an extremely unusual presentation. However, its close clinical and radiological similarity with malignancy makes it important in spite of its rarity. Here we report a case of bilateral lupus mastitis in male breast with its radiological features.

## Clinical presentation

A 39-year-old male presented with gradually increasing breast lumps on both sides for 1.5 years with no history of trauma. There was no past history of diabetes, hypertension, tuberculosis, pancreatitis, joint pains, fever or weight loss. On palpation, slightly tender subcutaneous masses measuring approximately 3 × 3  and 2 × 2 cm were noted in the left supra-areolar region and the right upper outer quadrant, respectively.

## Investigations

**Figure 1.**
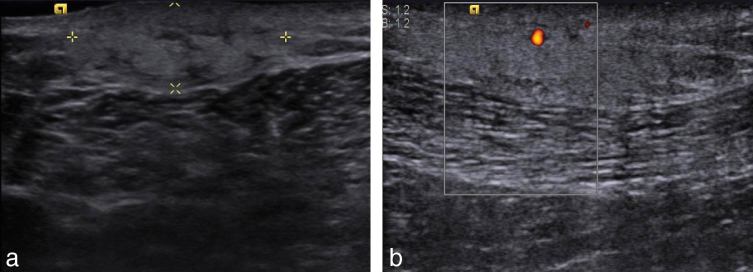
B-mode ultrasound (a) and Doppler (b) images of the left breast reveal a hyperechoic subcutaneous mass with internal vascularity.

Ultrasonography in the regions of the palpable abnormality (Figure 1) revealed an ill-defined, hyperechoic, subcutaneous mass with internal vascularity measuring 25 × 7 mm in the left supra-areolar region and another mass measuring 22 × 9 mm in the upper outer quadrant of the right breast [breast imaging-reporting and data system (BI-RADS) 4a]. Multiple subcentimetre lymph nodes with a few showing an attenuated hilum were seen in both the axillae (more on the left side) and the cervical regions.

**Figure 2.**
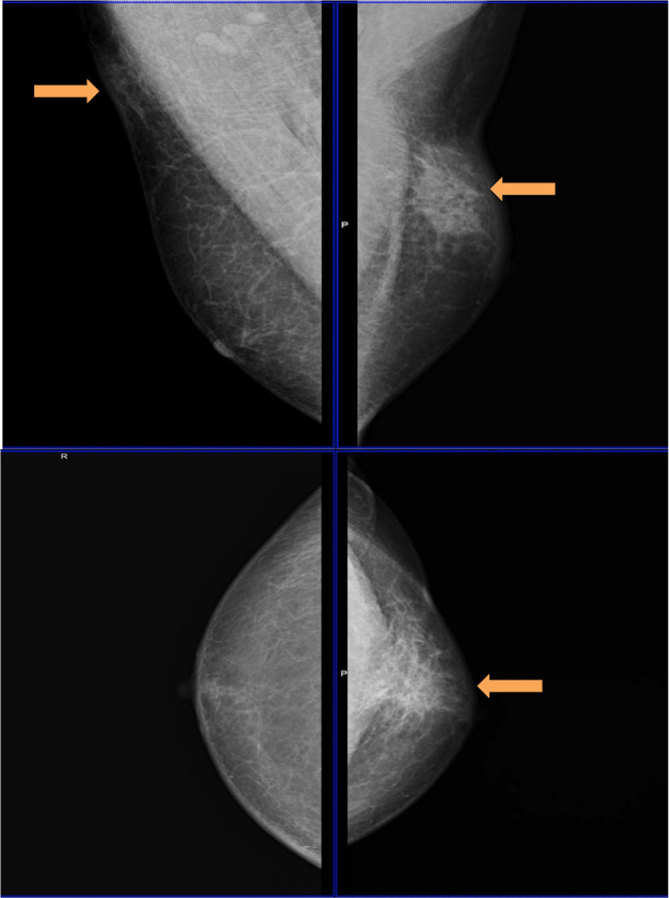
Mammogram of the right and left breasts showing ill-defined asymmetric masses (arrows) on both sides (BI-RADS 4a). The area of abnormality was not seen on the craniocaudal view of the right breast. BI-RADS, breast imaging-reporting and data system.

On mammography (Figure 2), an ill-defined, dense (compared with subcutaneous fat) mass was noted in the upper half of the left breast and upper outer quadrant of the right breast, with no evidence of calcification, architectural distortion or skin retraction (BI-RADS 4a).

**Figure 3.**
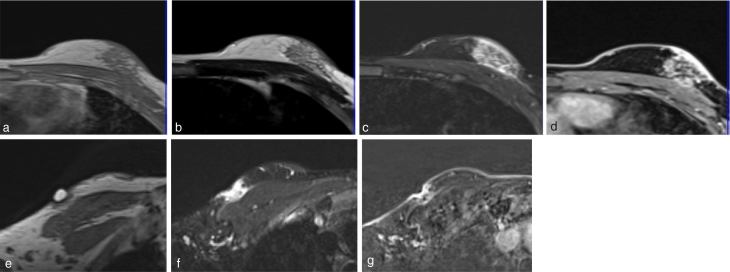
MRI of the left breast depicts isointense focal area of marked fat stranding with overlying skin thickening in the outer half of the left breast that is isointense on *T*_1_ (a) and hyperintense on *T*_2_ weighted (b) and STIR (c), and shows heterogeneous contrast enhancement (d). A lesion of similar signal intensity is seen in the right breast [*T*_1_ weighted (e) with a marker on the abnormal site, STIR (f) and contrast-enhanced *T*_1_ weighted (g)]. STIR, short tau inversion-recovery.

**Figure 4.**
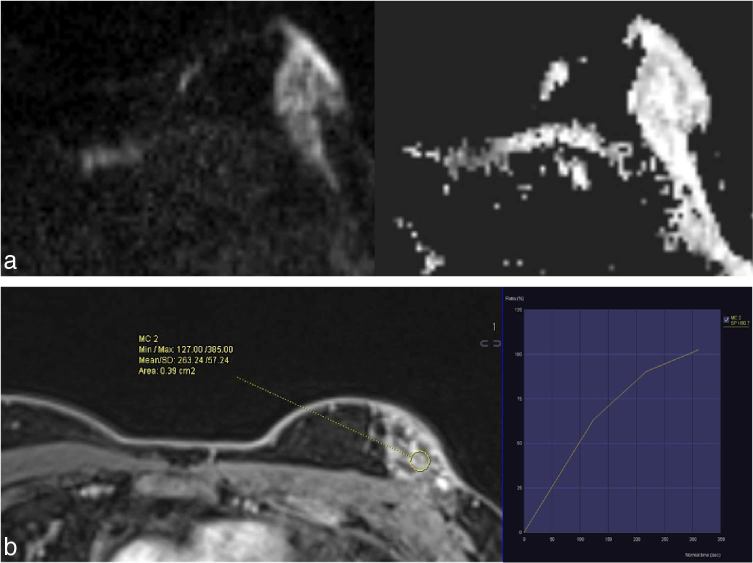
MRI of the left breast shows no evidence of restriction on diffusion-weighted imaging (a) and Type I kinetic enhancement curve (b).

Contrast-enhanced dynamic MRI of the breasts (Figures 3 and 4) showed focal areas of marked fat stranding with overlying skin thickening measuring 29 × 24 and 13 × 8 mm superolateral to the left nipple and the right upper outer quadrant, respectively. These areas were markedly hyperintense on *T*_2_ weighted fat-suppressed sequence. On contrast administration, the lesions showed heterogeneous enhancement and predominantly Type I kinetic enhancement curves. No evidence of restricted diffusion was noted bilaterally. Both the lesions had similar morphological and kinetic features. The lesions were characterized as BI-RADS 4.

**Figure 5.**
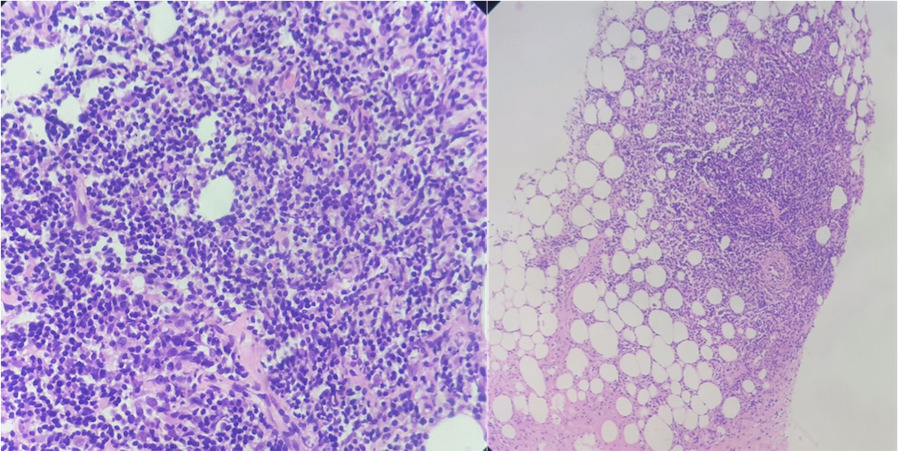
Histopathological images revealed fibrofatty tissue fragments infiltrated by lymphoplasmacytic cells arranged in a lobular and focally septal distribution.

Since the imaging findings were inconclusive, histopathological evaluation was advised. Ultrasonography-guided core needle biopsy was performed on the larger lesion in the left breast. Histopathology (Figure 5) revealed fibrofatty tissue fragments infiltrated by lymphoplasmacytic cells arranged in a lobular and focally septal distribution. Few of the vessels showed sclerosis, intimal oedema and lymphocytic infiltration. Focally, the infiltrate revealed a few scattered, intermediate-sized, transformed lymphocytes/immunoblasts. There was no evidence of granulomatous or neoplastic pathology. On immunohistochemistry (CD2, CD5 and CD7), no loss of T-cell antigen was seen. Histopathological findings were suggestive of panniculitis, likely autoimmune related.

Quantitative serum antinuclear antibody (ANA) analysis (IgG immunodot assay) was carried out and SS-A/Ro 60-kD, sp100, M2 recombinant and M2 native turned positive with values of 100, 33, 28 and 37 U ml^−1^, respectively. The ANA HEp-2 was positive at 1 :  40 titre and showed a fine speckled pattern. Anti-dsDNA antibody was negative. The leukocyte count, erythrocyte sedimentation rate, renal and hepatic function tests, urine analysis and serum amylase and lipase were within normal limits.

## Differential diagnosis

Fat necrosis, mastitis and cancer constituted the differential diagnosis that were considered in the work-up of the patient.

## Treatment and follow-up

**Figure 6.**
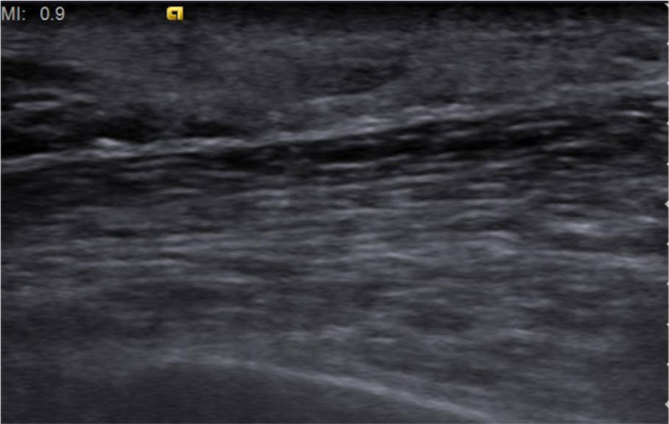
Follow-up ultrasound shows significant reduction in the size of the mass.

On the basis of histopathological and laboratory findings, the patient was diagnosed as a case of discoid lupus erythematosus (DLE) and treated with prednisolone and hydrochloroquine. On follow-up evaluation after 45 days, there was a significant reduction in pain and swelling. On ultrasonographical evaluation (Figure 6), there was a mild decrease in the size of the lesion.

## Discussion

Panniculus adiposus (subcutaneous fatty tissue) is composed of lobules and intervening septa containing vessels, lymphatics and nerves.^1^ Inflammation of subcutaneous fat “subcutaneous panniculitis” can be classified based on the aetiology, location and pathological pattern. However, it is difficult to differentiate these patterns on imaging.

Pathologically, it is broadly classified as lobular panniculitis with or without vasculitis, septal panniculitis with or without vasculitis and mixed panniculitis.^1,2^ Lobular panniculitis is seen in conditions such as systemic lupus erythematosus (SLE)/DLE, scleroderma, erythema induratum, trauma, neoplasms, inflammatory process, pancreatitis and Weber–Christian disease (idiopathic panniculitis). Septal panniculitis is seen in erythema nodosum, eosinophilic fasciitis and polyarteritis nodosa. Mixed panniculitis is seen in lupus profundus and subcutaneous sarcoidosis.^3,4^

Lupus is a condition of chronic autoimmune inflammation; when only the skin is involved, it is known as DLE, and when the internal organs are involved, it is called SLE.^5^

Lupus panniculitis or lupus erythematosus profundus occurs in 2–3% of patients with SLE or DLE.^6,7^ The term *lupus panniculitis* was proposed by Kaposi^8^ in 1883 and it usually occurs between 20 and 50 years of age, with female preponderance (female to male ratio of 2 : 1).^9^ Arms, buttocks, head, neck and thighs are the most commonly affected sites.^10,11^

Irgang introduced the term “lupus mastitis” for lupus panniculitis involving the breasts in 1940.^12^ Its coexistence is more common with DLE (33%) compared with SLE (10%).^10^ Until 2011, only 31 cases had been reported, out of which, 4 were in males; 19 in cases with SLE; 8 in cases with DLE; and in 4 cases, lupus was not categorized.^13^

Histopathological criteria for the diagnosis of lupus mastitis includes four major and minor criteria. The major criteria include hyaline fat necrosis, lymphocytic infiltration with lymphoid nodules surrounding the necrosis, periseptal or lobular panniculitis and microcalcifications. The minor criteria include discoid changes in overlying skin, lymphocytic vasculitis, mucin deposition, and hyalinization of subepidermal papillary lesions. Presence of all the criteria is not mandatory for diagnosis.^9,14^

Subcutaneous fat cells are destroyed, with subsequent haemorrhage and inflammatory infiltrate in the initial phase. This is followed by a reparative phase where fibroblasts proliferate, and in the final stage, fibrosis gradually replaces fat necrosis with little or no calcification.^15,16^

Lupus mastitis can mimic malignancy on both clinical examination and imaging. In our case, the masses were slightly tender, firm, non-mobile, subcutaneous, increasing in size, in the male breast with no acute signs of infection, ill-defined and hyperdense (compared with fat) on mammography, and ill-defined and hyperechoic to subcutaneous fat and showing increased vascularity on ultrasonography. These findings were of indeterminate aetiology.^2,11^ Findings on the mammogram and ultrasonography depend on pathological staging, and coarse calcifications become visible with disease progression.^11,15^

Pinho et al^16^ described the MRI features of non-necrotizing systemic granulomatous mastitis as lesions that were isointense on *T*_1 _and hyperintense on *T*_2_ weighted images with peripheral enhancing foci.Mosier et al^17^ also found delayed peripheral enhancement that showed discontinuous interval decrease in thickness with treatment. A predominant Type I kinetic curve was seen in the lesion in our case, which is concordant with findings of Pinho et al^16^ and discordant with findings of Sabaté et al^18^ who showed a Type III curve.Although, imaging findings are non-specific and cannot conclusively differentiate an inflammatory process from malignancy, an MRI is useful in assessing the extent of disease, presence of skin involvement and monitoring response to treatment.^17^

Differential diagnoses of lupus mastitis include idiopathic granulomatous mastitis that can also present with a similar clinical and imaging picture. But histologically, granulomatous mastitis is composed of lymphocytes, giant cells and plasma cells with occasional eosinophils. These features do not occur in lupus mastitis.^16^

Panniculitis-like T-cell lymphoma is very unusual but adds to significant clinical and imaging confusion. Although legs are the most common site of involvement, cases have been reported with breast involvement. Positive immunohistochemistry for T-cell lymphoma includes α/β T-cell receptor^+^, CD3^+^, CD4^–^, CD8^+^, CD56^–^, which were negative in our case.^19,20^

Panniculitis related to trauma or pancreatitis were not taken into consideration owing to the absence of history of trauma and normal serum amylase and lipase levels.

## Conclusions

Lupus mastitis or lupus panniculitis of male breast is a very uncommon condition that is a clinical and radiological mimicker of malignancy and in the absence of history of autoimmune disorder can lead to mismanagement of the disease as breast malignancy. The close clinical and radiological similarity of this condition with panniculitis-like T-cell lymphoma also adds to the diagnostic dilemma. However, histopathology, immunohistochemistry and serum markers for autoimmune disorders help in diagnosis.

## Learning points

1. Lupus mastitis is an unusual, benign disease that is very uncommon in males.
2. Imaging and clinical findings are non-specific as it mimics mastitis and malignancy.
3. Timely diagnosis with histopathological and serum analysis is the key to management.

## Consent

Written informed consent for the case to be published (including images, case history and data) was obtained from the patient for publication of this case report, including accompanying images and is within the hospital records.
